# Twin Pregnancy of a Complete Hydatidform Mole and a Co-existent Fetus: A Very Rare Case Report

**DOI:** 10.5812/ircmj.4244

**Published:** 2013-08-05

**Authors:** Afsaneh Tabandeh, Mahsa Besharat

**Affiliations:** 1 Golestan University of Medical Sciences, Gorgan, IR Iran; 2 Gastroenterology and Hepatology Research Center, Golestan University of Medical Sciences, Gorgan, IR Iran

**Keywords:** Moles, Pregnancy, Fetus

Dear Editor;

Molar pregnancy is a disorder of chorionic villi including trophoblastic proliferation and villous stromal swelling. One case of molar pregnancy is reported per 1500-2000 pregnancies in USA. Studies showed that the incidence of hydatiform mole in Asia, Africa and Central America is more than USA, Europe and Australia (1). Hydatiform mole is classified to partial (PM) and complete (CM) mole regards to the histopathology examination (2). Suspicion arises when an ultrasound reports a fetal pole associated with an abnormal placenta. (3) Twin pregnancy of a complete hydatiform mole and a natural fetus is a rare condition and accounts for one in 22000 to one in 100000 of pregnancies. Its importance is regards to the significant increased risk of pregnancy complications like preterm labor; making decision about the true management is much harder due to the probable viable fetus(3, 4).

In this paper we present a case of molar pregnancy with a co-existence abnormal fetus. A 23-years-old nulliparous woman was referred to our academic hospital with acute vaginal bleeding at 29th week of gestation, no pain and leaking was mentioned. She had a history of severe hirsotism and oligomenorrhea and previous hospitalization because of high blood pressure about two weeks ago (pre-eclampsia was ruled out at that time).Her husband had a family relation with her. In general appearance she had a marked edema in face and extremities. Fetal Heart rate (FHR) was134 beat/min and mother blood pressure (BP) was measured as 140/100 mmHg. She was diagnosed as having severe pre-eclampsia.

Laboratory tests were as followings:

Urea = 16 mg/dl, Creatinine = 0.8 mg/dl, Uric acid = 5.1 mg/dl, AST = 25 U/L, ALT = 29 U/L, Alk.P = 600 U/L, Hb = 12.2 gr/dl, HCT = 37%, platelet = 230000/microL, INR = 1, PT = 12 s, PTT = 33 s, Mother blood group = O^+^

Her ultrasound reported (at the 3rd month) molar changes and the second Ultrasound examination at this admission showed a hypervolumic placenta. Polycystic Ovary Syndrome (PCOs) was diagnosed regards to the clinical manifestations and laboratory testing. Pregnancy termination was suggested but she wanted to save the baby. Induction of labor with oxytocin was started, and she was transferred for cesarean section due to the arrested labor. Placenta showed molar changes in histopathologic examination and the female baby had a distended abdomen, severe bilateral hydronephrosis was reported in ultrasound due to the bilateral congenital polycystic kidney disease (PKD). Neonate was admitted to the pediatric referral hospital and a unilateral nephrostomy was done but she died after 3 days.

Twin pregnancy of a complete hydatidform mole and a co-existent fetus is one of the rare occurrences in the literature. Most symptoms are similar to a singleton hydatiform mole, including vaginal bleeding, failure of appropriate uterine growth and hypertension or pre-eclampsia in the first or second trimester (3). Some probable risk factors are mentioned in hydatidform mole but long term studies are needed to be confirmed (1). However, our patient had a history of oligomenorrhea and hirsotism and PCOs was diagnosed. None of the other studies we reviewed mentioned these symptoms neither the familial relationship between the parents. These could be hypothesized as probable risk factors.

Makrydimas et al reported a case of singleton pregnancy with a phenotypically normal fetus in which diffuse changes of complete mole were present in the placenta. The fetus was normal at birth and developed normally at 15 months (2). In our case, the fetus had abnormality in prenatal ultrasound and abdominal distension at birth and died after 3 days. Hamanoue et al also reported a twin pregnancy with complete hydatiform mole and preterm labor of a normal neonate (4). Other studies that reported twin pregnancy with hydatiform mole, mentioned a normal fetus such as Papoutsis et al, that reported a partial mole pregnancy with a chromosomically and phenotypically normal embryo, and Copeland et al who had reported dizygotic twin pregnancy with a normal fetus and partial hydatiform mole (5, 6). One of the noticeable facts in studies reviewed was the gender of the fetus which was female. Studies revealed a connection of partial moles with male fetus (1). It could be concluded that such cases of molar pregnancy as a twin to normal or abnormal fetus could be an area of interest in gynecology field and its etiology could be further studied.
